# Stiff-person syndrome in association with Hashimoto’s thyroiditis: a case report

**DOI:** 10.3389/fneur.2024.1360222

**Published:** 2024-07-17

**Authors:** Mingzhu Chen, Zhou Hong, Haicun Shi, Chunmei Wen, Yuan Shen

**Affiliations:** ^1^Department of Neurology, Affiliated Hospital 6 of Nantong University, Yancheng, Jiangsu, China; ^2^Department of Neurology, Yancheng Third People’s Hospital, Yancheng, Jiangsu, China; ^3^Department of Internal Medicine Neurology, Wuhan Fifth Hospital, Wuhan, Hubei, China

**Keywords:** stiff-person syndrome, Hashimoto’s thyroiditis, anti-GAD antibodies, autoimmune diseases, paraneoplastic syndromes

## Abstract

Stiff-person syndrome (SPS) is a rare neurological disorder characterized by chronic and progressive axial muscle rigidity and paroxysmal painful muscle spasms. The present case study described an SPS patient (increased anti-GAD65 antibody in serum and cerebrospinal fluid) with co-occurring Hashimoto’s thyroiditis and decreased C3 complement levels. The clinical presentation, diagnostic approach, and treatment employed for this unique case were comprehensively described in detail. In this case, we comprehensively presented a case of SPS with co-occurring Hashimoto’s thyroiditis and an associated decrease in serum C3 complement, as well as a discussion on the current data on this topic.

## Introduction

Stiff-person syndrome (SPS) was initially described as “stiff-man syndrome” by Moersch (1) and subsequently renamed by Blum and Jankovic (2). SPS is a rare autoimmune disorder characterized by chronic and progressive stiffness of the axial muscle or limbs, along with paroxysmal painful muscle spasms. The primary manifestation of SPS is the involvement of the nervous system, resulting in stiffness, weakness, and spasms in the trunk and limb muscles. Subsequently, an abnormal gait and impaired movement are observed in affected patients. Furthermore, laryngeal and respiratory muscle spasms can lead to breathing difficulties and esophageal obstruction, which may pose serious or even life-threatening consequences. Typical results of electromyography (EMG) include continuous motor unit activity (CMUA) in muscles at rest. Additionally, co-contraction of agonist and antagonist muscles is another sign observed on EMG. The onset of SPS typically occurs between the ages of 20 and 50 (3) and is frequently accompanied by some other autoimmune diseases, such as type 1 diabetes mellitus (T1DM) (4). Given the low incidence of SPS and limited reports on its concurrent occurrence with Hashimoto’s thyroiditis, along with decreased serum C3 complement levels, there may be a gap in the clinical understanding of SPS. In this case, we comprehensively presented a case of SPS with co-occurring Hashimoto’s thyroiditis and an associated decrease in serum C3 complement levels as well as a discussion on the current data on this topic.

### Case presentation

#### General case information

The patient is a 36-year-old right-handed Chinese married woman. The patient presented with initial symptoms of bilateral leg fatigue, difficulty initiating steps, and a loss of lower limb control. Subsequently, the patient’s symptoms gradually progressed to limb rigidity, limited knee joint mobility, and intermittent difficulty initiating movements. Eventually, both walking and standing functions were affected. At an early stage of the illness, the patient was diagnosed with a “somatic symptom disorder” at a major medical center according to the physical symptoms at the time. Subsequently, the patient’s condition evolved and progressed, with the manifestations of mental and psychological symptoms, such as anxiety, depression, paranoia, and obsessive-compulsive disorders. Accordingly, the patient was examined at a local neurology specialist hospital. Considering increased autoimmune thyroid antibody levels and the physical clinical symptoms, the patient was diagnosed with “Hashimoto’s thyroiditis and Hashimoto’s encephalopathy.” The patient’s symptoms improved with steroid treatment; however, discontinuation of steroids led to the recurrence of gait disturbance.

### Auxiliary examinations and investigations

**Figure 1:**
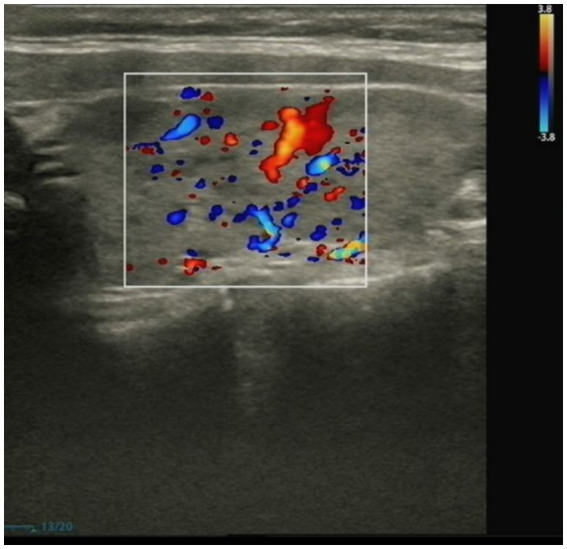
The representative ultrasonographic image of the thyroid of the patient.

**Figure 2:**
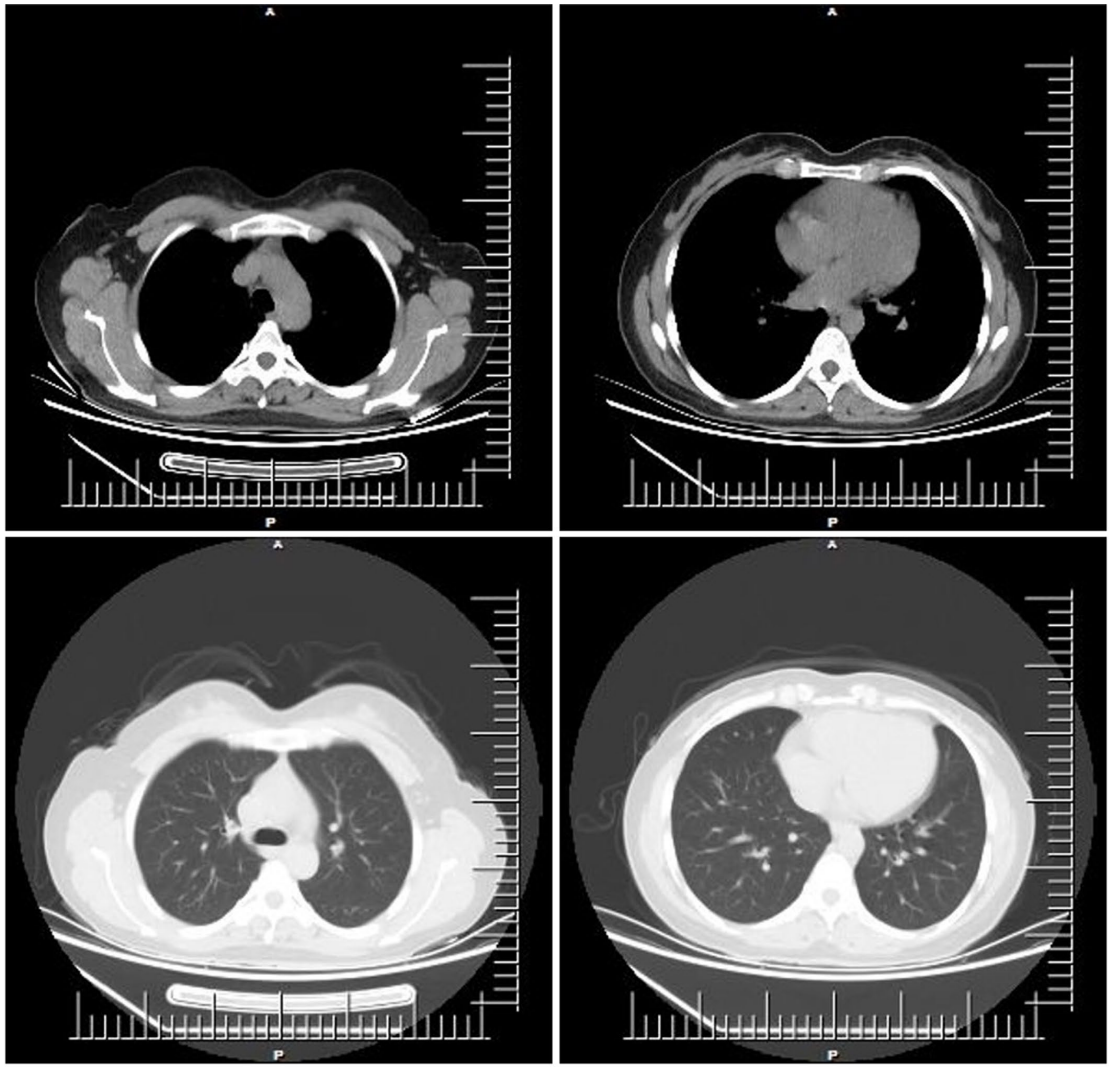
The representative chest computed tomography (CT) images of the patient.

**Figure 3:**
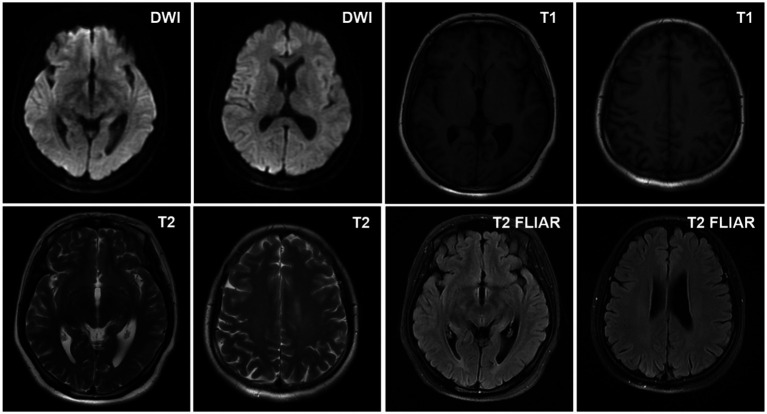
The representative images of brain magnetic resonance imaging (MRI) of the patient.

The patient mainly received routine blood tests, other serologic tests, and cerebrospinal fluid (CSF) tests, inducing creatine kinase, paraneoplastic autoimmune antibodies, antineutrophil cytoplasmic antibodies (ANCA), complement proteins, erythrocyte sedimentation rate, ceruloplasmin, folate, vitamin B12 (VitB12), tumor markers, rheumatoid factors, glycated hemoglobin (HbA1c), and thyroid function during the hospitalization (Supplementary Table 1). The elevated thyroid peroxidase and thyroglobulin antibodies indicated a thyroid-associated autoimmune disorder (Supplementary Table 2). Furthermore, within the spectrum of paraneoplastic antibodies, the presence of anti-glutamic acid decarboxylase 65 (anti-GAD65) antibody was positive, with serum titer of 100 AU and a CSF titer of 25 AU, which may have diagnostic implications (Supplementary Tables 3, 4). It was noted that there was a slight decrease in serum C3 complement levels but no change in C4 complement levels (Supplementary Table 5). Other parameters were within normal reference ranges. Additionally, the ultrasonography of the abdomen and pelvis revealed a slightly rough wall of the gallbladder and a cystic hypoechoic lesion in the left adnexal region in the patient. There were no obvious abnormalities or space-occupying lesions in the liver, pancreas, spleen, bilateral kidneys, bilateral ureteric ducts, bladder, or uterus. The ultrasonography of the thyroid showed heterogeneous changes in both thyroid glands (Figure 1). The chest computed tomography (CT) and brain magnetic resonance imaging (MRI) showed no significant abnormalities (Figures 2, 3).

**Figure 4:**
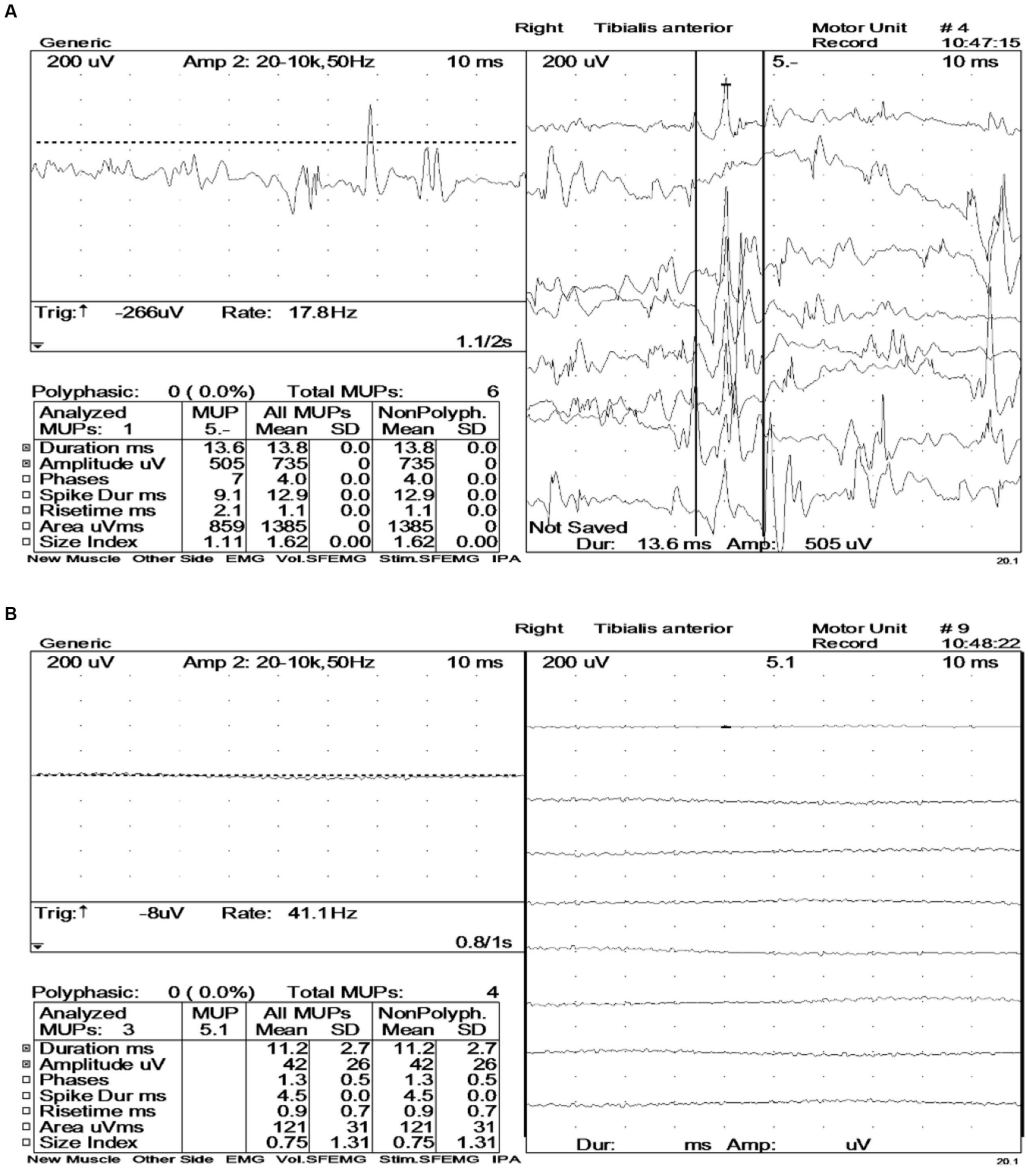
Electromyography (EMG) before and after intravenous administration of diazepam (10 mg). **(A)** Large numbers of motor unit-like units were seen in both the tibialis anterior muscles and the right vastus medialis muscle. During voluntary contractions, the duration and amplitude of the motor units in the examined muscles were all within the normal range. **(B)** During the relaxation phase, the right tibialis anterior muscle exhibited electrical silence, with no evidence of fibrillation potentials or positive sharp waves.

The diagnostic significance of EMG in the patient is markedly evident (Figure 4). Prior to the intravenous administration of 10 mg diazepam, extensive motor unit-like activity was observed in the muscles of both lower limbs, specifically the bilateral tibialis anterior and right vastus medialis (Figure 4A). Then, EMG recordings were taken at 1-, 3-, 5-, and 10-min intervals after diazepam administration, revealing that the right tibialis anterior muscle entered electrical quiescence during the relaxation phase without any signs of fibrillation potentials or positive sharp waves (Figure 4B). These findings strongly suggested a neuromuscular inhibitory effect induced by diazepam.

### Diagnosis process

The patient primarily presented with stiffness of the lower limbs and trunk, which could be triggered by tactile stimulation, such as mild contact with another individual. The serum and CSF testing yielded positive results for the anti-GAD65 antibody, and the treatment with clonazepam (a benzodiazepine) was proven to be efficacious. The EMG findings indicated significant motor unit potentials in the relaxed state of muscles in the lower limbs (bilateral tibialis anterior and right adductor magnus). However, these changes disappeared following the intravenous administration of diazepam. This case met the diagnostic criteria for SPS as published by Chia et al. (5).

### Case resolution

The patient initially received intravenous methylprednisolone at a dosage of 0.5 g per day for 5 days, followed by oral prednisone at a dosage of 40 mg per day, with a weekly reduction of 10 mg until 10 mg per day. Additionally, the patient was administered supplemental mycophenolate mofetil at a dosage of 0.25 g (bid), with an increment of 0.25 g per week until 0.5 g (bid) of mycophenolate mofetil. Furthermore, clonazepam (a daily dosage of 0.5 mg) and tizanidine were orally administered to the patient. Following this protocol, the patient experienced a significant improvement in limb stiffness and pain, along with increased ease in movement and the ability to climb stairs. Moreover, serum C3 complement levels returned to normal levels, while C4 complement levels remained unchanged (Supplementary Table 6).

## Discussion

The etiology and pathogenic mechanisms of SPS remain unclear. GAD65 is mainly localized in the gray matter of the spinal cord, basal ganglia, and brainstem nuclei. GAD65 antibody levels in the serum and cerebrospinal fluid of SPS patients are significantly elevated compared to healthy individuals (6). The potential mechanism underlying SPS involves an autoimmune response targeting the GAD65 protein within neurons, leading to a decrease in the synthesis and release of gamma-aminobutyric acid (γ-GABA) from inhibitory neurons. Decreased levels of γ-GABA may impede the formation of inhibitory signals in postsynaptic neurons, leading to a state of relative disinhibition among innervating nerves (7). This high excitability of lower motor neurons promotes continuous firing pulses that innervate skeletal muscles, ultimately causing muscle stiffness. Anti-GAD65 antibodies sometimes manifest in different clinical ways. The clinical manifestations of anti-GAD65 antibody-associated neuroimmune diseases are associated with the distribution of GAD65, with SPS, cerebellar ataxia, or limbic encephalitis as primary manifestations (8). Limbic encephalitis is the most common primary manifestation and frequently manifests as epileptic seizures, which is considered to be related to the inhibition of GABA function in hippocampal neurons and the excitability of the limbic cortex (9). Some patients exhibit stiff-person syndrome, which might be attributed to the hyperexcitability of α-motor neurons resulting from the inhibition of GABAergic cholinergic neurons in the notochord. This leads to the persistent synchronous contraction of antagonistic and non-antagonistic muscles (7). Additionally, the presence of cerebellar ataxia in some patients may be attributed to the inhibition of GABA release from cholinergic neurons in cerebellar Purkinje cells, leading to their excessive excitation (10).

SPS is an autoimmune disorder that may have co-occurring autoimmune disorders such as GAD-related T1DM, Hashimoto’s thyroiditis, Graves’ disease (GD), minimal change nephrotic syndrome, and myasthenia gravis (11) (Supplementary Table 7). Solimena et al. (12) initially identified the presence of GAD antibodies in the serum of a patient with SPS and T1DM, suggesting a potential intimate relationship between SPS and T1DM. We reported a case of SPS with Hashimoto’s thyroiditis. However, we observed a concurrent decrease in C3 complement levels in this case. The C3 complement is an active protein synthesized primarily by hepatocytes and macrophages within the complement system. It actively participates in both classical and alternative pathways of complement activation through its processes of activation and cleavage, thereby orchestrating the complement cascade and generating membrane attack complexes that ultimately result in cellular lysis. The activation of the complement system leads to decreased serum C3 complement levels. In systemic lupus erythematosus (SLE), lupus nephritis, and other related diseases, there is a consensus that C3 plays a crucial role in disease onset and progression. Some existing studies suggest that decreased C3 complement levels in patients with SLE are significantly related to disease activity, increased susceptibility to infection, and disease recurrence (13). Patients with lupus nephritis also exhibit decreased serum C3 complement levels, which can return to normal after remission. Studies also report that changes in C3 complement are associated with the development and progression of autoimmune diseases of the nervous system. A mouse model has demonstrated that selective inhibition of the C3a receptor can alleviate the degeneration of neurons in the brain of SLE mice. This may imply that the C3a receptor is a potential target of treatment for lupus encephalopathy (14). Previous studies have reported that activation of the complement system is associated with the pathogenesis of multiple sclerosis, and complement C3 may play a crucial pathogenic role in multiple sclerosis (15). Quantitative assessment of C3 uptake revealed that, in comparison to serum from healthy controls, serum from patients with Guillain–Barre syndrome exhibited a higher deposition of C3 fragments at sensitized targets (16). The findings of another study indicated the presence of C3d deposits in myelinated fibers within skin biopsies obtained from patients with paraproteinemic neuropathy (17). Furthermore, C3 complement deposits are also present in the neuromuscular junction of patients with myasthenia gravis, and exacerbation of myasthenia gravis is accompanied by increased complement consumption (18). These studies have indicated the association of C3 complement with autoimmune neurological diseases. In this case, we report a patient with SPS and Hashimoto’s thyroiditis who exhibited a reduction in complement C3 levels without any underlying etiologies accounting for such a decrease. Currently, there is a paucity of research on the immunological mechanism of SPS, and the role of C3 complement in SPS remains unknown. Studies have shown that the abnormal expression and activation of C3 complement can aggravate the damage to neurons in the brain. C3 complement binds to ligand-gated non-selective cation channels (such as transient receptor potential vanilloid type 1 (19)) on the cell membrane of neurons, triggering Ca^2+^ influx and inducing neuronal damage. A large number of C3 complement depletions cause a decline in the serum content of C3 complement. We detected decreased C3 complement levels in this SPS patient and observed a return to normal C3 complement levels 6 months after the treatment. Although there may be insufficient evidence to establish a definitive correlation between reduced C3 complement levels and SPS in the patient, this could present a possible opportunity to explore potential biomarkers for SPS. Additionally, it is important to note that complement activation plays a role in the pathogenesis of Hashimoto’s thyroiditis (20); therefore, we cannot definitively conclude that C3 complement alone contributes to the development of SPS. In other words, the decrease in C3 complement levels may also be a concurrent manifestation of Hashimoto’s thyroiditis. This limitation should be acknowledged in our report. In future studies, we will deeply focus on the alterations in C3 complement levels, particularly during immunotherapy, SPS disease recurrence, and Hashimoto’s thyroiditis remission or relapse.

The treatment options for SPS are diverse, with immunomodulatory therapy being the foremost and pivotal approach (21). High-dose corticosteroid pulse therapy is considered a potentially effective treatment for SPS. The patient exhibited significant symptom improvement following the administration of high-dose corticosteroid pulse therapy, thereby further substantiating the autoimmune etiology of SPS.

## Conclusion

The incidence of SPS in the population is relatively low, yet it poses significant health risks to patients. SPS is closely associated with autoimmunity, and the presence of anti-GAD65 antibodies is frequently detected in serum and cerebrospinal fluid. The present case study described a patient with SPS (increased anti-GAD65 antibody in serum and cerebrospinal fluid) co-occurring Hashimoto’s thyroiditis, accompanied by a decrease in C3 complement levels. The association between complement C3 and SPS, as well as its potential as a biomarker, requires further longitudinal observation in the future.

## Data availability statement

The data supporting the findings of this study are available from the corresponding author upon reasonable request.

## Ethics statement

The study involving a human was approved by the Ethics Review Committee of Yancheng Third People’s Hospital (Review-2023-59). The study was conducted in accordance with the local legislation and institutional requirements. The human samples used in this study were acquired from a by-product of routine care or industry. Written informed consent was obtained from the individual for the publication of any potentially identifiable images or data included in this article.

## Author contributions

MC: Writing – original draft, Data curation, Investigation, Validation. ZH: Data curation, Formal analysis, Writing – review & editing. HS: Data curation, Supervision, Writing – review & editing. CW: Data curation, Supervision, Writing – review & editing. YS: Conceptualization, Project administration, Writing – review & editing.
