# Supplementary material for: Stiff-person syndrome in association with Hashimoto’s thyroiditis: a case report
Source: Front Neurol. 2024 Jul 17;15:1360222. doi: 10.3389/fneur.2024.1360222 (PMC11290337; doi:10.3389/fneur.2024.1360222)
Supplement: Supplementary file 1 [file Data_Sheet_1.docx]

**Supplementary Table 1.** **The patient’s thyroid function test.**

| **Indicators** | **Serum levels** | **Reference range** | **Unit** |
| --- | --- | --- | --- |
| Thyroid stimulating hormone (TSH) | 2.630 | 0.27-4.2 | mIU/L |
| Free triiodothyronine (FT3) | 5.31 | 3.1-6.8 | pmol/L |
| Free thyroxine (FT4) | 14.70 | 12-22 | pmol/L |

**Supplementary Table 2.** **The patient’s thyroid autoantibody levels.**

| **Indicators** | **Serum levels** | **Reference range** | **Unit** |
| --- | --- | --- | --- |
| Thyroid peroxidase antibody (TPOAb) | > 600 | ≤ 34 | IU/mL |
| Anti-thyroid autoantibodies (TgAb) | > 4000 | ≤ 115 | IU/mL |
| Thyrotropin Receptor Auto-Antibodies (TRAb) | 1.74 | ≤ 1.75 | IU/L |

**Supplementary Table 3. Semi-quantitative results for paraneoplastic syndrome autoantibodies in serum of the patient.**

| **Antibody** | **Results** | **Detection method** | **Reference interval** |
| --- | --- | --- | --- |
| anti-Hu IgG antibody | Negative (< 5 AU) | BLOT | Negative: < 5 AU  Positive: > 10 AU |
| anti-Yo IgG antibody | Negative (< 5 AU) |  |  |
| anti-Ri IgG antibody | Negative (< 5 AU) |  |  |
| anti-CV2 (CRMP5) IgG antibody | Negative (< 5 AU) |  |  |
| anti-Amphiphysin IgG antibody | Negative (< 5 AU) |  |  |
| anti-Ma1 IgG antibody | Negative (< 5 AU) |  |  |
| anti-Ma2 IgG antibody | Negative (< 5 AU) |  |  |
| anti-Sox1 IgG antibody | Negative (< 5 AU) |  |  |
| anti-Tr (DNER) IgG antibody | Negative (< 5 AU) |  |  |
| anti-Zic4 IgG antibody | Negative (< 5 AU) |  |  |
| anti-GAD65 IgG antibody | **Positive (100 AU)** |  |  |
| anti-PKCy IgG antibody | Negative (< 5 AU) |  |  |
| anti-Recoverin IgG antibody | Negative (< 5 AU) |  |  |
| anti-Tintin (MGT30) IgG antibody | Negative (< 5 AU) |  |  |

Detection method for all antibodies is western blot (BLOT) unless otherwise specified.

**Supplementary Table 4. Semi-quantitative results for paraneoplastic syndrome autoantibodies in cerebrospinal fluid of the patient.**

| **Antibody** | **Results** | **Detection method** | **Reference interval** |
| --- | --- | --- | --- |
| anti-Hu IgG antibody | Negative (< 5 AU) | BLOT | Negative: < 5 AU  Positive: > 10 AU |
| anti-Yo IgG antibody | Negative (< 5 AU) |  |  |
| anti-Ri IgG antibody | Negative (< 5 AU) |  |  |
| anti-CV2 (CRMP5) IgG antibody | Negative (< 5 AU) |  |  |
| anti-Amphiphysin IgG antibody | Negative (< 5 AU) |  |  |
| anti-Ma1 IgG antibody | Negative (< 5 AU) |  |  |
| anti-Ma2 IgG antibody | Negative (< 5 AU) |  |  |
| anti-Sox1 IgG antibody | Negative (< 5 AU) |  |  |
| anti-Tr (DNER) IgG antibody | Negative (< 5 AU) |  |  |
| anti-Zic4 IgG antibody | Negative (< 5 AU) |  |  |
| anti-GAD65 IgG antibody | **Positive (25 AU)** |  |  |
| anti-PKCy IgG antibody | Negative (< 5 AU) |  |  |
| anti-Recoverin IgG antibody | Negative (< 5 AU) |  |  |
| anti-Tintin (MGT30) IgG antibody | Negative (< 5 AU) |  |  |

Detection method for all antibodies is western blot (BLOT) unless otherwise specified.

**Supplementary Table 5. Complement and immunoglobulins in serum of the patient.**

| **Indicators** | **Serum levels** | **Reference range** | **Unit** |
| --- | --- | --- | --- |
| Complement C3 | 0.865 | 0.9-1.8 | g/L |
| Complement C4 | 0.165 | 0.1-0.4 | g/L |
| IgA | 1.858 | 1.0-4.2 | g/L |
| IgG | 10.596 | 8.6-17.4 | g/L |
| IgM | 1.720 | 0.5-2.8 | g/L |

**Supplementary Table 6. Complement and immunoglobulins in serum of the patient after the treatment of six months.**

| **Indicators** | **Serum levels** | **Reference range** | **Unit** |
| --- | --- | --- | --- |
| Complement C3 | 1.001 | 0.9-1.8 | g/L |
| Complement C4 | 0.209 | 0.1-0.4 | g/L |
| IgA | 1.679 | 1.0-4.2 | g/L |
| IgG | 10.213 | 8.6-17.4 | g/L |
| IgM | 1.741 | 0.5-2.8 | g/L |

**Supplementary Table 7. Comparative literature case reports on Hashimoto’s thyroiditis-related SPS.**

| Coexisting diseases | Immunologic Markers | Treatment methods and effects | Refs |
| --- | --- | --- | --- |
| Graves’ disease (GD) | anti-GAD autoantibodies | Received a 5-day intravenous course of methylprednisolone at 1 g/d had little clinical improvement in her neurological condition received a course of intravenous immunoglobulin therapy (400 mg/kg daily), which yielded improvement in her painful spasms and gait, and she had no further falls | PMID: 28503622 |
| Chronic hepatitis C  Diabetes  Thyroid autoimmunity | GAD65-Abs  IgG1, IgG4 and IgE | No treatment regimen was involved. It is mainly described that interferon causes the occurrence of the above-mentioned diseases | PMID: 20541278 |
| Dysthyroid ophthalmopathy | anti-GAD autoantibodies | Therapeutic efficacy of plasmapheresis and high-dose intravenous immunoglobulin was transient. After starting administration of rituximab, the patient showed obvious improvement of muscle spasms due to stiff person syndrome Particularly in SPS in the present case, sudden onset spasms obviously improved soon after administration of rituximab, although the anti-GAD antibody remained at a high serum level. These findings suggest that the clinical effectiveness of rituximab in SPS and dysthyroid ophthalmopathy may be not only due to decreased concentrations of autoantibodies but also to other immune-mediated mechanisms | PMID: 20118602 |
| Autoimmune thyroiditis  Thymoma  Diabetes | anti-GAD and anti-amphiphysin autoantibodies | The patient experienced a partial improvement following a thymectomy and the administration of prednisone, intravenous immunoglobulins, and mycophenolate mofetil. Treatment with rituximab was followed by a complete sustained remission and the disappearance of serum anti-amphiphysin antibodies. | PMID: 20071184 |
| Acute ataxia  Graves’ disease (GD) | anti-GAD autoantibodies | The patient was given a trial of plasmapheresis over 3 days, and subsequently a 5-day course of intravenous immunoglobulin therapy (IVIG) at 0.4 g/kg/day with no improvement of her motor function. However, the immunosuppression produced a significant effect on her hyperthyroidism, within 2 weeks. | PMID: 17712846 |
